# Atlas of the Spectrum of a Platinum/Neon Hollow-Cathode Reference Lamp in the Region 1130–4330 Å

**DOI:** 10.6028/jres.097.002

**Published:** 1992

**Authors:** Jean E. Sansonetti, Joseph Reader, Craig J. Sansonetti, Nicolo Acquista

**Affiliations:** National Institute of Standards and Technology, Gaithersburg, MD 20899, USA

**Keywords:** hollow-cathode lamp, neon, platinum, spectral atlas, spectrum, wavelength

## Abstract

The spectrum of a platinum hollow-cathode lamp containing neon carrier gas was recorded photographically and photoelectrically with a 10.7 m normal-incidence vacuum spectrograph. Wavelengths and intensities were determined for about 5600 lines in the region 1130–4330 Å. An atlas of the spectrum is given, with the spectral lines marked and their intensities, wavelengths, and classifications listed. Lines of impurity species are also identified. The uncertainty of the photographically measured wavelengths is estimated to be ± 0.0020 Å. The uncertainty of lines measured in the photoelectric scans is 0.01 Å for wavelengths shorter than 2030 Å and 0.02 Å for longer wavelengths. Ritz-type wavelengths are given for many of the classified lines of Pt II with uncertainties varying from ±0.0004 to ± 0.0025 Å. The uncertainty of the relative intensities is estimated to be about 20%.

## 1. Introduction

The deployment of the Hubble Space Telescope (HST) on April 24,1990, launched a new era in astronomy. With the HST, stars and other astronomical objects are being observed with unprecedented clarity. The improvement over ground-based telescopes is most significant in the ultraviolet region of the spectrum, where the earth’s atmosphere absorbs most of the radiation. Although the much-publicized spherical aberration in the HST’s primary mirror [1] greatly reduces the quality of star images, many experiments of a spectroscopic nature are not severely affected because they do not require high spatial resolution. For example, for the Goddard High Resolution Spectrograph (GHRS), the highest resolution spectrograph on HST, the spherical aberration in the primary mirror does not degrade the spectral resolution noticeably when the small science aperture is used [2]. However, because of enlargement of the point spread function, the exposure time must be increased by a factor of about 5 to produce the signal-to-noise ratio of prelaunch expectations [2]. Nevertheless, spectra of very high quality have been obtained [2].

The region of observation of GHRS is 1100–3200 Å. In its echelle mode it has a resolving power of 90,000 and a wavelength accuracy of a few parts in 10^6^. Line-of-sight velocities of stellar objects can thus be determined to an accuracy of about 1 km/s. In order to achieve this accuracy, of course, an accurate wavelength scale must be established. This is accomplished by illuminating the spectrograph with an onboard platinum/neon hollow-cathode lamp during periods in which stellar observations are not being made [3]. The use of a Pt/Ne lamp for this purpose and its space-qualified design are due to Mount, Yamasaki, Fowler, and Fastie [4], who originally suggested it for wavelength calibration of the International Ultraviolet Explorer (IUE) satellite.

To achieve the accuracy for which GHRS was designed, the calibration wavelengths must be accurate to about 0.002 Å. However, tests carried out in our laboratory in 1983 indicated that the best available wavelengths for Pt [5] had errors ranging to about 0.015 Å. We thus began a program to measure the spectra emitted by a Pt/Ne hollow-cathode lamp similar to the one to be used with GHRS. This work was carried out with our high resolution 10.7 m normal-incidence vacuum spectrograph at NIST. At about the same time Engleman [6] recorded the spectrum of a Pt hollow-cathode lamp with a Fourier-transform spectrometer. He obtained accurate wavelengths for 320 lines of Pt I in the region 2200–7220 Å, optimized the energy level values, and calculated accurate Ritz-type wavelengths for 81 lines in the region 1724–2250 Å. Many of these lines were used in calibrating our grating measurements.

Some of the results of our work have appeared in two previous papers. In the first [7] we determined accurate values for 100 energy levels of Pt II by combining our new grating measurements for over 500 Pt II lines in the ultraviolet with measurements of lines at longer wavelengths made by Engleman by Fourier transform spectroscopy. In the second [8] we reported wavelengths with accuracies of 0.002 Å or better for some 3000 lines emitted by a Pt/Ne lamp in the region 1032–4100 Å. In this second report we also provided relative intensities of the spectral lines of the Pt/Ne lamp that were determined by recording the spectra photoelectrically with the same spectrograph used for the wavelength measurements.

Our wavelengths for the Pt/Ne lamp are currently being used for calibration of GHRS as well as for wavelength calibration of the Faint Object Spectrograph on HST, which uses a Pt-Cr/Ne hollow-cathode lamp for both wavelength and radiometric calibration [9]. Our data are also being used for revised calibrations of spectra from the IUE satellite [10], and for calibration of spectra obtained with sounding rockets, which also use onboard Pt/Ne hollow cathode lamps [11]. In a different type of application, the data are being used to interpret the spectra of stars that contain Pt in anomalously high abundances [12].

In the present paper we present a comprehensive report of our observations of the Pt/Ne hollow-cathode lamp. For completeness we give a full account of the experimental work and data analysis. Some of this information has been given in our previous papers.

Our results are presented in the form of an atlas of the spectrum emitted by a Pt/Ne hollow-cathode lamp in the region 1130–4330 Å. The atlas consists of plots of the spectrum accompanied by tables that include the wavelengths, wave numbers, intensities, and identifications or classifications where known for more than 5600 lines. We have attempted to provide the best available wavelength data, substituting values from the literature or calculated Ritz-type wavelengths where these are more accurate than our measurements.

The line list developed in this work was communicated to J. Blaise and J.-F. Wyart of the Laboratoire Aimé Cotton, Orsay, France, who have used it to substantially extend the energy level analysis of Pt II. Based on our measurements they have located nearly 150 new Pt II levels. Their report on the analysis appears as a companion paper in the same issue of this journal [13]. Blaise and Wyart have also located about 100 new levels of Pt I. The new line identifications for Pt I and II have been provided to us and are incorporated in the atlas.

The data included in this atlas should be of use not only for astronomical spectroscopy but also for the calibration of general laboratory spectra obtained with medium to high resolution diffraction grating spectrographs. No other source provides such a dense and complete coverage of this spectral region with lines suitable for use as reference wavelengths. The Pt/Ne hollow cathode is easy to operate and is commercially available at moderate cost.

## 2. Photographic Observations

Our observations were made with the 10.7 m normal-incidence vacuum spectrograph at the National Institute of Standards and Technology. Two different gratings were used, the first blazed at 1200 Å in first order and the second blazed at 3000 Å in first order. Both gratings were ruled with 1200 lines/mm. All measurements were made in the first order, the plate factor being 0.78 Å/mm. The slit width was 0.023 mm. With this slit width the resolving limit throughout the region of observation was about 0.020 Å. Photographic exposures were made on Kodak SWR plates.^1^

Two different light sources were used. The first was a windowless, demountable hollow-cathode lamp having a solid copper cathode containing a helical platinum wire and some chips of silicon and germanium. The general design of the lamp was similar to that of Reader and Davis [14]. In the version used in the present work the O-ring assembly at the front of the lamp was replaced by a large ball joint by which the lamp could be connected directly to the spectrograph. The lamp was operated in series with a 300 Ω ballast resistor at a dc voltage of 250 V and a current of 90 mA. The cathode was cooled with flowing water. The carrier gas consisted of flowing helium with a trace of neon at a total pressure of approximately 266 Pa (2 Torr). With this gas mixture the spectra of both Cu and Pt could be excited simultaneously. This could not be accomplished when only a single gas was used. Exposure times for this lamp were about 15 min.

The second source was a sealed hollow-cathode lamp similar to the one used by GHRS. It has a platinum hollow cathode with neon carrier gas and is sealed with a magnesium fluoride window. The lamp was manufactured by the Westinghouse Corporation (Model WL34045). It was connected to the spectrograph by a quick-disconnect flange. The cathode was located 215 mm from the slit. The lamp was operated with a 5000 Ω ballast resistor at a dc voltage of 310 V and a current of 20 mA. Exposure times ranged from 2 to 150 min.

In the first phase of the wavelength reductions of the photographic data, the spectra of Pt observed with the demountable Pt-Cu lamp were measured with respect to lines of Cu II, Si I, Si II, Ge I, Ge II, Ne I, and Ne II to determine accurate wavelengths for a select group of Pt lines. Wavelengths for Cu II were Ritz values derived from the level values of Ross [15]. Wavelengths for most Ne I and II lines above 2780 Å were taken from the Fourier-transform measurements of Palmer and Engleman [16]. Wavelengths for other Ne II lines above 2780 Å and all Ne II lines below this wavelength were Ritz values given by Persson [17]. Ne I, Si, and Ge wavelengths were taken from the compilation of reference wavelengths by Kaufman and Edlén [18]. The measurements made with the demountable Pt-Cu lamp provided accurate values for about 1500 lines of Pt I and II extending from 1032 to 2885 Å.

In the second phase of the reductions the spectra of all lines observed with the sealed Pt/Ne lamp were measured with respect to the above group of Pt lines, lines of Ne I and II, and lines of Pt I reported by Engleman [6]. In the region above 2885 Å, our reference spectra consisted solely of lines of Ne I, Ne II, and Pt I with wavelengths taken from the sources cited above.

Next, our values for lines of Pt II with known classifications were combined with values for classified lines of Pt II measured by Engleman by means of Fourier-transform spectroscopy to determine accurate values for 28 even and 72 odd energy levels of Pt II [7]. Using these level values we calculated Ritz-type wavelengths for almost all of the classified lines of Pt II. For some of these levels the energy or *J* value has been revised as a result of the work of Blaise and Wyart [13]. For those levels that have not been changed, the Ritz values have been substituted for the measured values in the final list of wavelengths.

## 3. Photoelectric Observations

To determine the relative intensities of lines emitted by the Pt/Ne lamp and to observe lines weaker than those recorded on the photographic plates, we recorded the spectrum by translating an exit slit and photomultiplier tube along the focal curve of the 10.7 m vacuum spectrograph. The entrance and exit slit widths were 0.050 mm. The line intensities were measured by photon counting. Signals from the photomultiplier were amplified and processed by a discriminator and logarithmic ratemeter. The analog output signal from the ratemeter was sampled at 1 Hz by a computer, which digitized and stored the data. This acquisition rate corresponded to a wavelength interval of 0.0086 Å per sample. Prior to each scan the analog response of the ratemeter was calibrated by using a pulse generator to simulate the amplified pulse signal from the photomultiplier tube. The response of the ratemeter was digitized and recorded for pulse frequencies ranging from 10/s to 10^6^/s by decades.

The resolution limit for the scans was about 0.07 Å. The spectrum was scanned in overlapping 650 Å segments, each segment corresponding to a different rotational setting of the grating. Each scan lasted 20 h. Two scans were made for each region above 1685 Å, the first a normal scan and the second a scan at reduced sensitivity to record very intense lines that were saturated at normal recording conditions. The sensitivity was reduced by introducing a one decade offset in the logarithmic ratemeter. In addition, for the region above 2000 Å, the source intensity was attenuated by reflecting the lamp from an uncoated glass plate.

Four different Pt/Ne lamps were used in the course of the experiments. Two lamps were used for the photographic exposures. One of these and two additional ones were used for the photoelectric scans. The longest use of any lamp was during the photoelectric scans, where one of the lamps was run for about 250 h. After this time the cavity of the cathode had become noticeably enlarged.

The position and intensity of each spectral line in the photoelectric scans was determined by using a computer line-finding algorithm. First, the recorded signal at each point in the spectrum was converted to absolute counts/s by using the calibration information mentioned above. Then these data were scanned by the computer to locate peaks in the spectrum. The position of each peak was determined by calculating the quadratically smoothed first derivative of the data in the vicinity of the maximum intensity point and linearly interpolating the zero crossing of the derivative. The wavelength was then calculated by making a linear fit of wavelength versus position for the local spectral region, using as standards four lines accurately measured from the photographic observations on either side of the line to be determined.

The intensities derived from the raw data for each scan were adjusted to produce a consistent set of values over the whole spectral region. First, using the measured intensities for lines of moderate strength in the overlapping regions of the various scans, a set of multiplicative factors was determined to bring the separate scans onto the same relative scale. Then the spectral response of the spectrograph/detector combination as a function of wavelength was calibrated by using accurate radiance values for about 80 lines of platinum measured by Klose [19] in a similar Pt/Ne hollow-cathode lamp. All of the spectral data were corrected for this instrumental response. Thus the intensities plotted in the atlas are on a true relative scale.

The number of lines observed by photon counting was much greater than observed photographically. Whereas the weakest photographic lines produced count rates of about 500 photons/s, lines having signals as low as about 10 photons/s could be observed photoelectrically. The most intense lines produced counts of about 2,000,000 photons/s. In all scans we observed a residual background count in excess of the photomultiplier dark count. This background was only a few counts/s at low wavelengths but increased to about 60 counts/s at the highest wavelengths. This increasing background is apparent in the atlas plots. The background count has been subtracted from the measured line intensities printed in the table so that the value reported accurately reflects the count rate due to the spectral line.

## 4. Description of the Atlas

The atlas is a series of tables and plots that provides a comprehensive description of the spectrum of the Pt/Ne hollow-cathode lamp in the region 1128–4333 Å. Each page of plots depicts a 32 Å section of the spectrum. Every spectral line for which a wavelength and intensity have been determined is indicated with a tic mark at the bottom of the plot. The wavelengths, wave numbers, and relative intensities for these lines are listed in the table on the page facing the plot.

The wavelengths and intensities of Rowland ghosts (spurious lines caused by imperfections in the ruling of the grating) were predicted from the known properties of the gratings. Ghost lines are marked on the plots with a carat instead of a tic mark to distinguish them from true spectral lines. They are not listed in the table.

Wavelengths of lines measured on our photographic plates, taken from the literature, or calculated from optimized Pt II energy levels are given to four decimal places. Lines measured in the photoelectric data only are given to two decimal places. Wavelengths below 2000 Å are given in vacuum; wavelengths above 2000 Å are given in air. For lines originally observed in vacuum, conversion of the wavelengths from vacuum to standard air was carried out by using the three-term formula of Peck and Reeder [20] for the index of refraction of air.

Also listed in the table under the column heading CODE are the sources for wavelengths of various lines emitted by the Pt/Ne lamp that we have taken from the literature, mainly Pt I, Ne I, and Ne II. Most of these lines were used as wavelength standards. Literature values were also substituted for lines of impurity species such as H I, C I, O I, Si I, Al I, and Al II. The presence of additional impurity lines of Mg I, Mg II, Fe I, Cr I, Pd I, Rh I, Au I, Ag I, Ni I, Ca I, and Ca II were subsequently pointed out by J. Blaise. These lines are identified in the table. Literature values for their wavelengths have been substituted only for Ca II and Fe I.

The intensity of impurity lines varies greatly from lamp to lamp. For example, we did not observe the intense Al I lines at 3944 and 3961 Å on our photographic plates. However, in a lower wavelength exposure using a different lamp the normally less intense lines at 3082 and 3092 Å did appear. For this reason we have given no intensities for the impurity lines.

The energy level designations for classified lines of Pt I and II correspond to the integer parts of the level energies and are given with the even parity level first. Classifications and wavelengths for Pt I lines with CODES D and E were taken from Engleman [6]. Pt I lines with CODE N and Pt II lines with CODE K are newly classified by Blaise and Wyart [13]; the wavelengths are from the present work. Classifications for other Pt II lines were taken from Shenstone [5], with level values given by Reader, Acquista, Sansonetti, and Engleman [7]; a number given in the CODE column is the wavelength uncertainty of the Ritz wavelength in units of 0.0001 Å (see Sec. 5).

The intensities in the atlas are a uniform set of relative values covering the entire region of observation. For lines that were blended on the photoelectric scans but resolved or nearly resolved on the photographic exposures, the intensities were estimated visually from the photographic plates by comparison with nearby well-resolved lines. In a few places a real spectral line is blended with a grating ghost. This is noted with an M in the CODE column in the table. The intensities measured for such lines are probably affected by the presence of the ghost. As mentioned, the spectral sensitivity of the spectrometer and detector combination was taken into account by using the accurate radiance values of Klose [19] for about 80 of the lines to normalize the observations. From the reproducibility of our measurements and comparisons with the data of Klose we estimate the relative intensities for a given species (element and stage of ionization) to be accurate to about 20%. A prime factor in possible variation of the relative intensities is the length of time that a particular lamp has been used. Over many hours of use the intensities of the Ne lines are observed to change relative to the Pt lines. However, for a given atom and ionization stage the relative intensities should be reliable within our estimated uncertainty. For most lines the present intensities are identical to those given by Reader, Acquista, Sansonetti, and Sansonetti [8]. The intensities of a few lines have been slightly revised in the present work.

Our relative intensities for lines emitted by the Pt/Ne lamp are potentially useful for calibration of the spectral response of spectrographic systems in other laboratories. In general, the values are sufficiently reliable to provide a good semi-quantitative calibration. Of course the accuracy that can be obtained is limited by the degree to which other Pt/Ne lamps might vary from those we used. We found only small variations in the relative intensities of lines in our lamps, all of which were purchased separately over a 5 year period. Nevertheless, it is not certain that other lamps would exhibit identical properties. In particular, comparison of lines in the 1130–1300 Å region with lines in higher wavelength regions could be affected by variation in the low wavelength transmission of the magnesium fluoride windows of different lamps. Since we used only a small number of lamps and did not scan each lamp over the entire spectral region, we can make no definitive statement regarding lamp to lamp variation. Further investigation would be needed to evaluate the importance of such systematic variations.

## 5. Accuracy of Wavelengths

Our estimate of the uncertainty of the photographically measured wavelengths is based on several considerations:

- The standard deviation of our polynomial fits for the Cu II reference lines in the Pt/Cu lamp was typically 0.0010 Å.
- The standard deviation of our polynomial fits for the Pt lines used as internal standards for measurements in the Pt/Ne lamp was typically 0.0015 Å.
- A comparison of a group of about 100 lines measured by different operators on different plates and taken with different grating rotations in the region 1470–1520 Å showed an average deviation of 0.0001 Å and an rms difference of 0.0014 Å. In general, our separate measurements of the wavelengths of individual lines agreed to about this level of accuracy.
- A comparison of the wavelengths of 37 lines of Pt II in the region 2247–3700 Å that were measured in this work and independently by Engleman [7] shows an average deviation of 0.0003 Å and an rms difference of 0.0019 Å.
- For the 508 lines of Pt II whose wavelengths can be calculated from the optimized level values, the rms difference between the calculated and observed wavelengths is about 0.0015 Å.
- A comparison of our measured wavelengths for impurity lines appearing in the Pt/Ne lamp with standard wavelengths for these lines shows an average deviation of 0.0003 Å and an rms difference of 0.0015 Å.

Based on these comparisons we estimate an uncertainty of ± 0.0020 Å for the wavelengths measured photographically.

As mentioned above, the wavelengths of classified lines of Pt II in the atlas which have numbers in the CODE column are those derived from the optimized level values. The uncertainties of these wavelengths are taken to be the square root of the sum of the squares of the uncertainties of the combining levels as given by Reader, Acquista, Sansonetti, and Engleman [7]. They are listed in the far right column under the heading CODE in units of 0.0001 Å.

The uncertainties of the photoelectrically measured lines were estimated by comparing the measured wavelengths of Pt II lines observed only in the photoelectric scans with calculated Ritz wavelengths for the same lines. The standard deviation of the differences was about 0.006 Ä for lines below 2030 Å and about 0.015 Å for lines at longer wavelengths. Based on these comparisons we estimate the uncertainty to be ±0.01 Å for lines below 2030 Å and ±0.02 Å for lines above 2030 Å.

The uncertainties of lines whose wavelengths have been taken from the literature are discussed in some detail in the notes to the atlas. Most of these uncertainties are less than 0.001 Å and virtually all are less than 0.002 Å.

The cathodes of the lamps used in this work and with GHRS contain isotopes of Pt in their natural abundances. Some lines of Pt I and II show appreciable isotope and magnetic hyperfine structure (hfs). At the resolution of our spectrograph (and also GHRS) almost all Pt lines appear sharp and symmetric. A few lines show evidence of unresolved structure and appear wide, hazy, or asymmetric on the photographic plates. These lines are noted (W, H, L, or S) adjacent to their intensities in the atlas. Lines showing partially resolved structure are noted in the atlas as being complex (C). A few hyperfine patterns occurred in the photographic data as three fully resolved features and were measured as separate lines.

For GHRS and other instruments with resolving power of 10^5^ or less, the existence of hfs in some lines should present no problem in using the present list of Pt lines for wavelength calibration. To achieve the highest accuracy, lines with notations indicating detectable unresolved structure should not be used. For instruments with resolving limits significantly below 0.02 Å, structure may be observed in many additional Pt lines, and our present wavelength list may not be adequate for calibration purposes. Thus, for calibration of spectrographs having much higher resolution, it may be desirable to develop calibration wavelengths based on a lamp whose cathode contains a single even isotope of Pt.

## Footnotes

1 Certain commercial equipment, instruments, or materials are identified in this paper to specify adequately the experimental procedure. Such identification does not imply recommendation or endorsement by the National Institute of Standards and Technology, nor does it imply that the materials or equipment identified are necessarily the best available for the purpose.
